# Association of Obesity-Related Genetic Variants with Android Fat Patterning and Cardiometabolic Risk in Women

**DOI:** 10.3390/genes16091019

**Published:** 2025-08-28

**Authors:** Débora Sá, Maria Isabel Mendonça, Francisco Sousa, Gonçalo Abreu, Matilde Ferreira, Eva Henriques, Sónia Freitas, Mariana Rodrigues, Sofia Borges, Graça Guerra, António Drumond, Ana Célia Sousa, Roberto Palma dos Reis

**Affiliations:** 1 Centro de Investigação Dra. Maria Isabel Mendonça, Hospital Dr. Nélio Mendonça, SESARAM EPERAM, Avenida Luís de Camões, nº 57, 9004-514 Funchal, Portugal; drmendonca@sesaram.pt (M.I.M.); fhgsousa@sesaram.pt (F.S.); goncalobabreu@sesaram.pt (G.A.); matilde.ferreira@sesaram.pt (M.F.); eva.henriques@sesaram.pt (E.H.); soniafreitas@sesaram.pt (S.F.); mariana@sesaram.pt (M.R.); sofia.borges@sesaram.pt (S.B.); maria.taipa@sesaram.pt (G.G.); anacelia.sousa@sesaram.pt (A.C.S.); 2 Serviço de Cardiologia, Hospital Dr. Nélio Mendonça, SESARAM EPERAM, Avenida Luís de Camões, nº 57, 9004-514 Funchal, Portugal; cardiologia@sesaram.pt; 3 Laboratório de Genética Humana, Universidade da Madeira, 9020-105 Funchal, Portugal; 4 NOVA Medical School, Faculdade de Ciências Médicas, Campo dos Mártires da Pátria 130, 1169-056 Lisboa, Portugal; palma.reis@nms.unl.pt

**Keywords:** obesity, android obesity, genetic variants, diabetes, cardiometabolic risk

## Abstract

**Background/Objectives:** The location and distribution of excess fat, rather than overall adiposity, are stronger predictors of cardiometabolic risk and are commonly assessed using the waist-to-hip ratio (WHR). Fat distribution in women has a heritable component, yet the genetic factors that influence it remain poorly understood. We aim to assess the association between obesity-related polymorphisms with WHR and cardiometabolic risk in overweight and obese women. **Methods:** A cohort study was conducted in 512 women (56.1 ± 6.4 years; body mass index (BMI) ≥ 25 kg/m^2^). WHR was calculated, and participants were classified into android (WHR > 0.85) or gynoid (WHR ≤ 0.85) obesity groups. We genotyped 15 SNPs previously associated with obesity using TaqMan real-time PCR. Different genetic models (dominant, recessive, and allelic) were analysed, and bivariate and multivariate analyses were performed to compare the fat distribution groups. **Results:** Of the 15 SNPs studied, only 3 presented a significant association with WHR > 0.85. *PSRC1* rs599839 in a dominant model (AA + GA vs. GG) with OR = 3.18 (*p* = 0.041), *SLC30A8* rs1326634 in a recessive model (CC vs. TC + TT) (OR = 2.38; *p* = 0.004), both showing increased susceptibility to central obesity. *KIF6* rs20455 offers protection in a recessive model (CC vs. TC + TT) with an OR of 0.47 (*p* = 0.043). After adjusted multivariate analysis, only *SLC30A8* and diabetes remained independently associated with an increased risk of android obesity (OR = 2.50; *p* = 0.003 and OR = 3.63; *p* = 0.004, respectively). **Conclusions**: The *SLC30A8* variant was significantly associated with android fat distribution and high cardiometabolic risk in overweight/obese women. Identifying genetic factors that influence fat distribution may help specify targeted lifestyle changes or pharmacological interventions to reduce risk.

## 1. Introduction

Overweight and obesity are well-established risk factors for cardiovascular disease (CVD) and cardiometabolic disease (CMD). Although obesity prevalence in some high-income countries appears to have stabilised, it remains at markedly elevated levels. In contrast, low-income nations continue to experience a sustained rise in obesity rates, and many middle- and high-income regions are now facing an escalating epidemic of severe obesity (BMI ≥ 35 kg/m^2^) [1].

Body mass index (BMI) can serve as a marker indicating general obesity. Four categories of BMI are in everyday use today to estimate patient situations: underweight or undernourished status (BMI < 18.5 kg/m^2^); normal BMI (18.5–24.9 kg/m^2^); overweight (25.0–29.9 kg/m^2^); and obesity (BMI ≥ 30 kg/m^2^) [2]. BMI provides only an indirect estimate of body fat and may misclassify individuals with greater muscle mass. It does not account for the aetiology or heterogeneity of obesity, nor does it capture variations in outcomes across individuals or populations. Despite these limitations, BMI continues to be a practical, inexpensive screening tool in clinical practice, although its restricted value for individualised risk prediction is well recognised.

Epidemiological studies suggest that the location and distribution of excess fat, rather than overall adiposity, provide better insights into the risk of cardiometabolic diseases. Fat distribution can be assessed through the waist-to-hip ratio (WHR), which is calculated by dividing the waist measurement by the hip measurement. WHR offers a more accurate reflection of intra-abdominal fat accumulation and can better predict the occurrence of CVD than BMI and waist circumference (WC). Previous systematic reviews and meta-analyses have demonstrated that WHR is an excellent predictor of myocardial infarction risk, and it is more effective in women than in men [3]. Independent of their overall BMI, individuals with higher central adiposity have an increased risk of insulin resistance [4], metabolic syndrome [5], diabetes, and stroke [6].

WHR is also an independent determinant of infertility, with values > 0.85 associated with a higher risk compared to WHR ≤ 0.85 [7,8].

Fat distribution has been shown to have a heritable component, with twin-based heritability estimates ranging from 30% to 60% [9,10] and a narrow-sense heritability estimated at approximately 50% in women and around 20% in men. In the polymorphic obesity type, common genetic variants, usually with small effect sizes, contribute to the risk of developing obesity and cardiometabolic disease and can then be further modulated by environmental factors [7]. However, interactions between common and rare genetic factors and environmental/lifestyle factors modulate the risk of developing obesity and cardiometabolic disease [11].

Given that obesity increases the risk of developing critical diseases in life, specifically in women, a better understanding of the genetic basis of obesity will lead to early lifestyle changes and other interventions in the genetically predisposed population.

Our study aimed to assess the association between obesity-related polymorphisms with waist-hip ratio (WHR) and lipid–metabolic risk in overweight and obese women.

## 2. Methods

### 2.1. Study Design and Population

Five hundred and twelve women with BMI ≥ 25 kg/m^2^ (overweight and obese women) with a mean age of 56.1 ± 6.4 years were selected from the Madeira Island population. Written informed consent was obtained from all at the time of enrolment. We conducted a case–control study in which the cases were women with WHR > 0.85, and the controls were women of similar ages with WHR ≤ 0.85. This work was conducted following the Declaration of Helsinki and was approved by the Ethics Committee and Institutional Council of the Funchal Hospital Centre, under protocol number 50/2012.

### 2.2. Assessment of the Cardiovascular Risk Factors

Demographic, traditional, and clinical risk factors such as age, smoking status, physical inactivity, obesity, arterial hypertension, type 2 diabetes mellitus (T2DM), dyslipidaemia, and CAD family history were inquired in a presential clinical appointment, as previously described [12]. For the WHR calculation, the waist circumference was measured at the narrowest point between the lower rib and the iliac crest, while the hip circumference was measured at the widest part of the hips. WHR was obtained by dividing the waist circumference by the hip circumference.

Participants were then classified into two groups based on their waist-to-hip ratio (WHR) values: central obesity, characterised by a WHR > 0.85, and gynoid obesity, characterised by a WHR ≤ 0.85.

### 2.3. Biochemical Risk Factors

Blood samples were extracted after 12 h of fasting. Biochemical analyses were performed in the hospital’s Central Laboratory using standard techniques.

To determine total cholesterol, HDL, LDL, glucose, and triglycerides, blood samples were placed in dry tubes and centrifuged 30 min later at 3500 g. They were subsequently quantified by an enzymatic technique using an auto-analyser AU 5400 (Beckman Coulter, Brea, CA, USA). Biochemical markers, such as lipoprotein (a) (Lp(a)) and apolipoprotein B (Apo B), were quantified by immunoturbidimetry using an automatic AU 5400 system (Beckman Coulter). Homocysteine was measured by fluorescence polarised immunoassay using an Abbott IMX automatic device (Abbott Laboratories, IL, USA).

### 2.4. Genetic Analysis

In the present study, we analysed 15 SNPs previously associated with obesity in Genome-Wide Association Studies (GWAS) or Candidate Gene Studies. These genes are part of the GENEMACOR Case–Control study developed in the Madeira Population [13]. The genetic variants analysed in this work are described in the supplementary Table S1.

Genotyping was performed in peripheral leukocyte DNA using the TaqMan genotyping assay (Applied Biosystems, Foster City, CA, USA) with allelic discrimination. Labelled probes and primers pre-established by the supplier (7300 Real-Time PCR System, Applied Biosystems, USA) were used without prior knowledge of the individual’s clinical data. The quality of the genotyping technique was verified by including one non-template control in each 96-well plate and a blind duplicate, which accounted for 20% of all samples.

### 2.5. Statistical Analysis

#### Descriptive and Comparative Analysis

Continuous variables are presented as mean values with standard deviations or as medians with their respective ranges, according to distribution. Categorical variables are summarised as absolute frequencies and percentages. Comparisons between continuous variables were performed using either Student’s *t*-test or the Mann–Whitney *U* test, depending on data distribution, while categorical variables were compared using the χ^2^ test. To assess associations between genotypes and demographic, biochemical, or clinical characteristics, bivariate analyses with the χ^2^ test were conducted. We tested for Hardy–Weinberg equilibrium and explored genetic models to detect different genetic effects, which may provide insights into the strength and nature of the association.

Bivariate and multivariate logistic regression analyses investigated the association between SNPs and WHR, adjusted for traditional risk factors. We explored different genetic models (dominant, recessive, or allelic) to study the best genetic model for comparison. Other genetic models provide insight into the strength of the association. We consider the best model to be the one that yields the most significant effect in strengthening the association analysis. After multivariate analysis, we assessed the biochemical profile of the variants independently associated with central fat distribution through 2D dot plots. All analyses were performed using SPSS version 25 (SPSS Inc., Chicago, IL, USA). Statistical significance was set at a 5% level (*p* < 0.05).

## 3. Results

### 3.1. Main Characteristics of the Population (Overweight and Obese Women) According to Fat Phenotype (Android or Gynoid)

The population’s primary demographic, clinical, and laboratory parameters are reported in Table 1 and compared between women with a waist-to-hip ratio (WHR) greater than 0.85 and those with a WHR of 0.85 or less. Women with android obesity were significantly more likely to have type 2 diabetes mellitus (T2DM) (*p* = 0.003), higher fasting glucose levels (*p* = 0.001), higher triglyceride levels (*p* = 0.001), and lower HDL-cholesterol levels (*p* = 0.021) than women with gynoid fat distribution (Table 1).

### 3.2. Genetic Variants Associated with Obesity (Bivariate Analysis)

**Figure 1:**
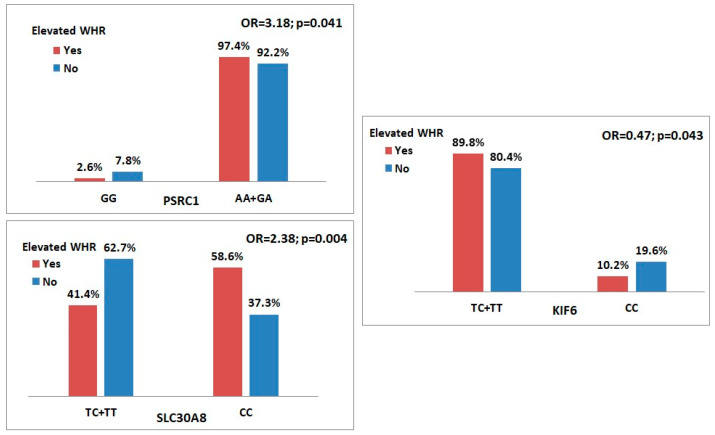
Genotype distribution of overweight and obese women according to fat phenotype. Bar chart of genotype percentages for the SNPs associated with central/android obesity [*PSRC1* rs599839 (G > A), *SLC30A8* rs1326634 (T > C)]—genetic risk and [*KIF6* rs20455 (T > C)]—protection.

Of the 15 genetic variants previously studied for association with obesity, only 3 showed an association with central or android-type fat distribution after bivariate analysis. These were all in Hardy–Weinberg equilibrium, as shown in Table 2. Two SNPs showed an increased risk, namely *PSRC1* rs599839 in the dominant model (AA + GA vs. GG) with an odds ratio (OR) of 3.18 (95% CI 0.99–10.27; *p* = 0.041) and *SLC30A8* rs1326634 in a recessive model (CC vs. TC + TT) with an OR of 2.38 (95% CI 1.31–4.33; *p* = 0.004). The *KIF6* rs20455 in the recessive model (CC vs. TC + TT) showed significant protection (OR = 0.47; 95% CI 0.22–0.99; *p* = 0.043) (Table 2 and Figure 1).

#### Multivariate Analysis

In the multivariate logistic regression analysis, the following variables were included: *SLC30A8* (CC), *PSRC1* (AA + GA), *KIF6* (CC), diabetes, age, smoking, hypertension, dyslipidaemia, BMI, and physical inactivity. Among these, only diabetes (OR 3.63, *p* = 0.004) and *SLC30A8* rs1326634 (CC vs. TC + TT) (OR = 2.50; *p* = 0.003) remained in the equation as independently associated with an increased WHR, that is, with the central distribution of fat (android phenotype) (Table 3).

### 3.3. Biochemical Profile of the Genetic Variant SLC30A8 (CC) in a Cohort of Overweight and Obese Women

#### 3.3.1. Fasting Glucose Profile

**Figure 2:**
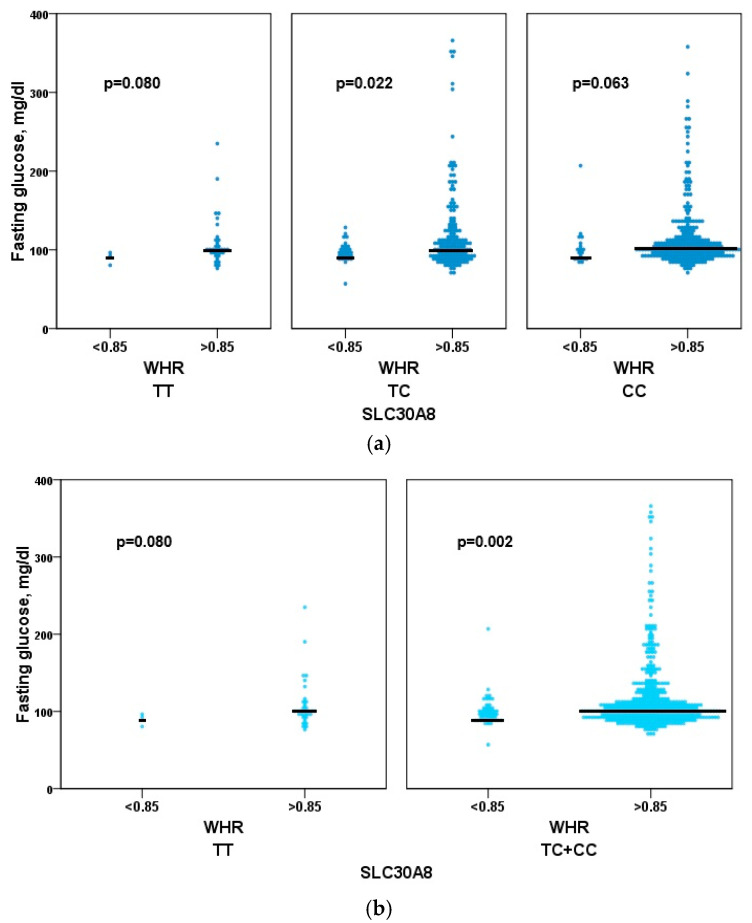
Association of *SLC30A8* rs1326634 with fasting glucose. Associations of the functional variant in the *SLC30A8* gene (rs1326634) with fasting glucose levels in the women’s cohort. We used the three genotypes CC, TC, and TT (**a**) or the dominant genetic model (**b**) (CC + TC and TT) to compare the two different fat distributions (WHR > 0.85 and WHR ≤ 0.85).

The C risk allele was significantly over-represented in the women with WHR > 0.85 (75.7%) than in those with WHR ≤ 0.85 (65.7%). The heterozygous genotype (TC) in women with a waist-to-hip ratio (WHR) greater than 0.85 was significantly associated with higher median glucose levels compared to the WHR ≤ 0.85 group (*p* = 0.022). There was also an association in the homozygous CC women’s group, but with marginal significance (*p* = 0.063). Only the dominant genetic model (CC + TC) in the group with WHR > 0.85 was significantly associated with higher median fasting glucose levels when compared with WHR ≤ 0.85 (*p* = 0.002) (Figure 2).

#### 3.3.2. Lipid Profile

##### Triglyceride

**Figure 3:**
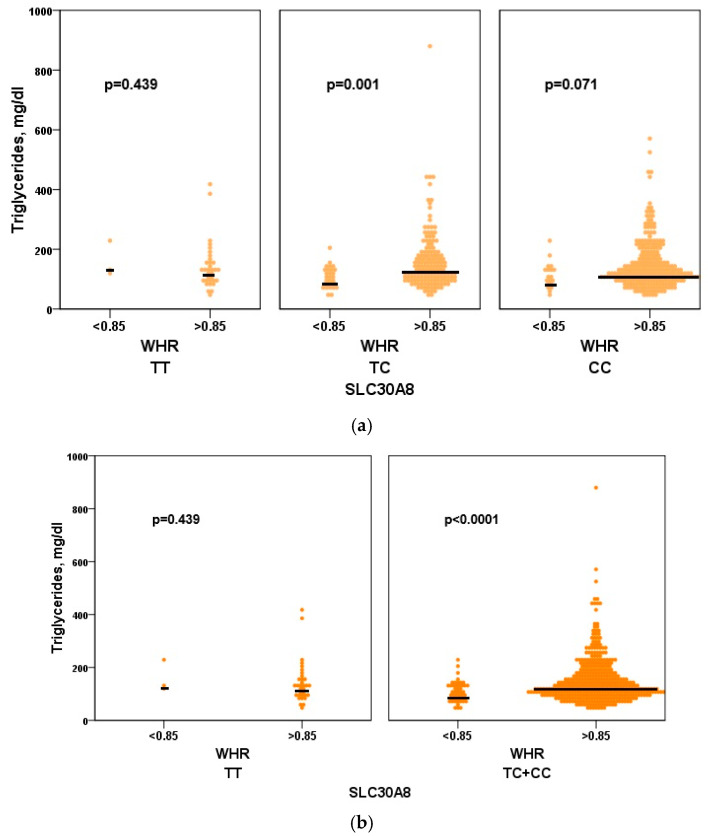
Association of *SLC30A8* rs1326634 with triglycerides. Associations of the functional variant in the *SLC30A8* gene (rs1326634) with triglyceride levels in the female cohort. We used the three genotypes CC, TC, and TT (**a**) or the dominant genetic model (**b**) (CC + TC and TT) to compare the two different fat distributions (WHR > 0.85 and WHR ≤ 0.85).

The two genotypes, heterozygous (TC) and homozygous (CC), in the group with a waist-to-hip ratio (WHR) greater than 0.85, were associated with higher median levels of triglycerides compared to those with a lower WHR. However, the TC genotype yielded significant results (*p* = 0.001), while the CC genotype only showed marginal significance (*p* = 0.071). The dominant genetic model (CC + TC) in the women’s cohort with WHR > 0.85 revealed the best association with higher median triglyceride levels (*p* < 0.0001) (Figure 3).

##### HDL Cholesterol

**Figure 4:**
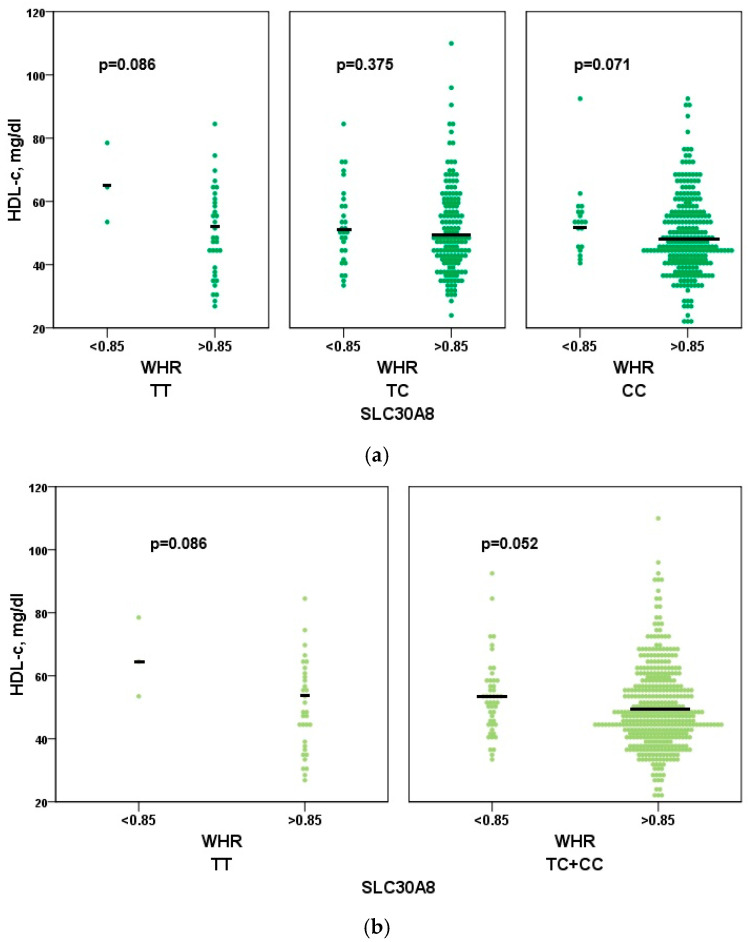
Association of SLC30A8 rs1326634 with HDL-c. Associations of the functional variant in the *SLC30A8* gene (rs1326634) with HDL cholesterol levels in the female cohort. We used the three genotypes CC, CT, and TT (**a**) and the dominant genetic model (**b**) (CC + TC and TT) to compare the two different fat distributions (WHR > 0.85 and WHR ≤ 0.85).

The wild genotype (TT) and homozygous (CC) genotypes in android obese women were associated with lower median levels of HDL cholesterol compared with the group with a waist-to-hip ratio (WHR) ≤ 0.85, but it did not reach statistical significance (*p* = 0.086 and *p* = 0.071, respectively). The dominant genetic model (CC + TC) was associated with lower HDL cholesterol levels, approaching statistical significance (*p* = 0.052) (Figure 4).

Only the best genetic model (dominant model) demonstrated a statistically significant association between this model and metabolic biochemical alterations [increased fasting glucose (*p* = 0.002) and hypertriglyceridemia (*p* < 0.0001)] in women with a WHR > 0.85 compared to those with a WHR ≤ 0.85. The association of low HDL cholesterol values and *SLC30A8 rs1326634* (dominant model) almost reached statistical significance (*p* = 0.052) in WHR > 0.85 compared to ≤0.85.

## 4. Discussion

Obesity is a complex, multifactorial condition whose pathogenesis involves biological, psychosocial, socioeconomic, and environmental determinants, as well as heterogeneous mechanisms and pathways that contribute to adverse health outcomes [14].

As previously mentioned, fat accumulation in specific body regions, particularly in the abdominal area, is linked to a heightened risk of metabolic and cardiovascular conditions, as well as increased mortality. WHR serves as a simple and accessible indicator of body fat distribution. A higher WHR suggests a greater amount of intra-abdominal fat and is associated with an elevated risk for diabetes and cardiovascular disease. In contrast, a lower WHR indicates a greater proportion of fat stored in the gluteal region and is associated with a reduced risk of diabetes, hypertension, dyslipidaemia, and mortality. Subcutaneous fat distribution, particularly in the lower body, is metabolically protective [15].

A clear sexual dimorphism exists in the prevalence of obesity, attributable to differences in its pathophysiology, including patterns of fat distribution, sex hormone profiles, energy metabolism, and gut microbiota composition, as well as chromosomal and genetic influences [16]. GWAS have identified over 250 common genetic variants associated with body mass index (BMI), a simple index generally used to indicate obesity. However, obesity is a heterogeneous disease, and research must move beyond using BMI as a sole measure. Genetic studies focused on specific adiposity-related traits are expected to identify additional loci, thereby uncovering novel biological mechanisms [17]. Many factors influence fat distribution and show sexual dimorphic effects, meaning they have stronger associations in women than in men. This fact supports that oestrogen and other sex hormones modulate gene expression and adipose tissue function differently in women [18,19,20]. A recent genome-wide meta-analysis involving 224,459 individuals identified 49 loci associated with BMI-adjusted waist-to-hip ratio, including 33 novel loci and additional loci linked to waist and hip circumference. Notably, 20 of these *loci* exhibited significant sexual dimorphism, with 19 showing more potent effects in women. The associated loci were enriched in genes expressed in adipose tissue and adipocyte regulatory elements. Pathway analyses highlighted the roles of adipogenesis, angiogenesis, transcriptional regulation, and insulin resistance in influencing fat distribution, shedding light on the biological mechanisms underlying body fat patterning [21].

In this study, we used a list of 15 polymorphic genes previously connected with general obesity in the GENEMACOR study. After exhaustive consultation of the literature, of the 15 listed, only 4 genes, *FTO* SNP rs9939609 T > A, *MC4R*, SNP rs17782313 T > C, *PPARG* rs1801282 C > G, and *ADIPOQ* rs2241766, rs1501299, have been previously explicitly and independently linked to the waist-to-hip ratio after BMI adjustment.

In our study, of this genetic list, only three variants showed association with this central obesity distribution type: *PSRC1* rs599839 G > A, which encodes a protein involved in the leptin–melanocortin pathway regulating appetite and energy balance; *SLC30A8* rs1326634 T > C, generally associated with T2DM and obesity; and *KIF6* rs20455 T > C variant, which, in this study, showed a lower susceptibility to obesity (protection) to this type of android fat phenotype. These data suggest that the *KIF6* rs20455 variant may protect against obesity susceptibility in this sex-specific group. Recent research has identified another *KIF6* variant, rs9471077, which has been associated with an increased risk of obesity in males [22]. However, further research is needed to confirm these associations and understand the underlying mechanisms.

After adjusting for confounding variables in the multivariate logistic regression analysis, only *SLC30A8* rs1326634 remained independently associated with central obesity. Women carrying this SNP have a 150% higher probability of developing central obesity.

*SLC30A8* (Solute Carrier Family 30 Member 8) is a gene codifying a zinc transporter 8 (ZnT8), a protein critical in shuttling zinc into insulin granules in pancreatic β-cells. Variants in the *SLC30A8* gene can impair insulin secretion and action, key features in T2DM and insulin resistance, which are precursors to obesity. ZnT8 is highly expressed in pancreatic beta cells, localises to insulin secretory granules (ISGs), and regulates zinc content, which is essential for insulin synthesis, storage, and secretion. This variant can disrupt insulin secretion and impair insulin’s action, which are key features of T2DM, as previously reported in multiple studies on T2DM [23,24,25,26]. Other studies have also reported an association with T2DM complications like retinopathy and neuropathy [27]. More recent research in mice has shown that the global knockout of ZnT8 results in severe insulin resistance and obesity. Altered zinc homeostasis can disrupt adipokine signalling, promote inflammation, lead to dysfunctional fat storage, and be linked to the development of obesity. Both genetic polymorphisms and environmental factors affecting zinc homeostasis may contribute to the development of obesity and related metabolic disorders [28].

The relationship between obesity and T2DM has long been recognised and clearly explains the high prevalence of T2DM achieved in many countries. Obesity is frequently accompanied by hypertension and dyslipidaemia, leading to a clustering of metabolic and cardiovascular risk factors. It is also a key contributor to metabolic alterations such as T2DM. Accordingly, several studies have shown a progressive increase in CVD risk with higher BMI values.

Advances in imaging technologies have transformed the study of body adiposity and its associations with health outcomes. Due to the distinct properties of fat, muscle, and bone tissues, it is now possible to assess body composition and body fat partitioning phenotypes with precision. Imaging studies revealed that the amount of adipose tissue in the abdominal cavity, also known as visceral adipose tissue (VAT), was a key correlate of the health risk associated with a large waistline for a given body mass index (BMI). Moreover, when individuals with similar BMI were compared based on their VAT content, those with higher visceral fat accumulation exhibited a more adverse cardiometabolic profile. This unfavourable profile was predictive of a greater likelihood of developing T2DM and cardiovascular complications, regardless of their overall body weight or percentage of body fat [29].

The rs13266634 (T > C) polymorphism in the *SLC30A8* gene has been extensively studied in type 2 diabetes mellitus (T2DM), with the C allele consistently associated with an increased risk of disease. However, its relationship with obesity or patterns of fat distribution is less common. A 2009 study in a Cuban American population found that individuals with the CC genotype of the *SLC30A8* rs13266634 had a 2.11-fold higher likelihood of presenting with central Obesity than TC carriers, suggesting that the C allele is associated with increased disease risk in this population. Nevertheless, other studies have not found significant associations between this variant and central obesity [30]. This ambiguity and the limited evidence available further highlight the relevance of our study.

*SLC30A8* rs1326634 is a genetic variant in our study that is independently associated with the female android obesity phenotype. The cellular mechanisms and physiopathology underlying the linkage between obesity and T2DM are multifaceted and involve adiposity-induced alterations in β-cell function, sometimes linked to common genetic defects, adipose tissue biology, and insulin resistance, which are often ameliorated or even normalised with adequate weight loss. The zinc metabolism influences adipocyte differentiation and function. Altered zinc homeostasis can disrupt adipokine signalling, promote inflammation, and lead to dysfunctional fat storage, all of which are linked to the development of central obesity. It is, therefore, not surprising that these two conditions, T2DM and central obesity, share common polymorphic variations [31].

Regarding the analysis of the biochemical markers, our findings indicate that the SLC30A8 risk allele (C) is associated with adverse metabolic traits predominantly in women with central fat distribution (WHR > 0.85). In this subgroup, carriers of the allele, particularly under the dominant genetic model, exhibited higher fasting glucose and triglyceride levels, with a strong trend towards lower HDL cholesterol. These results suggest that the impact of SLC30A8 variants may be modulated by fat distribution, amplifying metabolic risk when adiposity is predominantly visceral. While SLC30A8 has been primarily studied in the context of glucose homeostasis and type 2 diabetes, our data extend its possible role to lipid dysregulation, highlighting a broader contribution to the metabolic disturbances that characterise android obesity. Although exploratory, these observations raise the hypothesis that genetic susceptibility conferred by SLC30A8 may interact with central obesity to exacerbate cardiometabolic risk, a relationship that deserves further investigation in larger cohorts.

### Strengths and Limitations

In terms of strengths, the present work showed that a simple genetic analysis (*SLC30A8* rs1326634) may serve as a genetic tool for identifying a higher propensity for central obesity in women. No robust, direct WHR association has been found in the available scientific literature to date. This finding makes our work the first to have found a strong association with this cardiometabolic index. The presence of this genetic marker may alert to early cardiometabolic alterations that lead to an increased risk of major cardiovascular events. This indicator may serve as guidance for early reinforcement of lifestyle changes and careful monitoring of glycolipid levels, which are relevant to primary prevention.

Fat distribution is regulated by genetic variation (SNPs) but is influenced by ethnicity and environmental factors. In the present study, individuals of other ethnicities were not investigated; only Caucasian individuals from southern European populations were included, which can be considered a study limitation.

## 5. Conclusions

*PSRC1* rs599839, *SLC30A8* rs1326634, and *KIF6* rs20455 genetic variants showed association with central fat distribution in our female cohort. However, a significant and independent risk association was found only between the *SLC30A8* rs1326634 variant and android/central fat distribution in women. Alterations in zinc transport mechanisms, such as those involving this variant and the ZnT8 transporter, can contribute to zinc metabolic disturbances that lead to type 2 diabetes mellitus and obesity. Understanding the genetic basis of obesity and central fat distribution in women can enable lifestyle changes or pharmacologic interventions to attenuate the risk of cardiometabolic complications.

## Figures and Tables

**Table 1:** Baseline characteristics of overweight and obese women.

Variables	Total (*n* = 512)	WHR > 0.85 (*n* = 461)	WHR ≤ 0.85 (*n* = 51)	*p* Value
Age, years	56.1 ± 6.4	56.1 ± 6.5	56.7 ± 6.4	0.540
Smoking, n (%)	91 (17.8)	84 (18.2)	7 (13.7)	0.425
AHT, n (%)	352 (68.8)	322 (69.8)	30 (58.8)	0.107
CAD FH, n (%)	118 (23.0)	105 (22.8)	13 (25.5)	0.662
T2DM, n (%)	151 (29.5)	145 (31.5)	6 (11.8)	**0.003**
BMI ≥ 30 kg/m^2^, n (%)	229 (44.7)	211 (45.8)	18 (35.3)	0.153
Dyslipidaemia, n (%)	435 (85.0)	396 (85.9)	39 (76.5)	0.074
Physical inactivity, n (%)	293 (57.2)	265 (57.5)	28 (54.9)	0.724
EAT, cm^3^	6.1 (2.3–12.0)	6.2 (2.3–12.0)	5.8 (2.4–8.6)	0.159
Fasting glucose, mg/dl	102.0 (57.0–366.0)	103.0 (70.0–366.0)	96.0 (57.0–208.0)	**0.001**
Apo B, mg/dl	92.4 (3.9–199.3)	92.4 (3.9–171.1)	90.2 (6.2–199.3)	0.433
Hcy, mg/dl	11.2 (2.9–48.7)	11.1 (2.9–48.7)	11.9 (4.0–32.0)	0.415
Lp (a), mg/dl	15.5 (0.9–241.0)	15.5 (0.9–241.0)	15.0 (3.0–179.2)	0.982
Triglycerides, mg/dl	124.0 (42.0–880.0)	127.0 (42.0–880.0)	109.0 (42.0–231.0)	**0.001**
TC, mg/dl	192.0 (98.0–341.0)	193.0 (98.0–323.0)	185.0 (131.0–341.0)	0.378
LDL-c, mg/dl	115.3 (15.6–236.4)	115.3 (15.6–236.4)	105.5 (58.2–211.8)	0.650
HDL-c, mg/dl	48.0 (21.7–110.0)	47.0 (21.7–110.0)	53.0 (33.0–92.0)	**0.021**
Non-HDL-c, mg/dl	144.0 (59.0–269.0)	145.0 (59.0–269.0)	130.9 (83.0–258.0)	0.143

WHR—waist-to-hip ratio; AHT—arterial hypertension; CAD—coronary artery disease; FH—family history; T2DM—type 2 diabetes mellitus; BMI—body mass index; EAT—epicardial adipose tissue; Apo B—apolipoprotein B; Hcy—homocysteine; Lp (a)—lipoprotein a; TC—total cholesterol; LDL-c—low-density lipoprotein cholesterol; HDL-c—high-density lipoprotein cholesterol; continuous variables presented by mean ± SD or median (min–max); statistically significant at *p* < 0.05.

**Table 2:** Genotype distribution according to fat phenotype of the overweight and obese women.

Genetic Variants	WHR > 0.85 (*n* = 461)	WHR ≤ 0.85 (*n* = 51)	Odds Ratio (95% CI)	*p* Value	HWE *p* Value
*PSRC1* rs599839 (G > A)					0.057
G	188 (20.4)	30 (29.4)	1.63 (1.03–2.56)	0.035	
A	734 (79.6)	72 (70.6)			
Genotype					
GG	12 (2.6)	4 (7.8)	Reference		
GA	164 (35.6)	22 (43.1)	2.49 (0.74–8.38)	0.131	
AA	285 (61.8)	25 (49.0)	3.80 (1.14–12.66)	0.020	
Best model					
Dominant (AA + GA vs. GG)			3.18 (0.99–10.27)	0.041	
*SLC30A8* rs1326634 (T > C)					0.448
T	224 (24.3)	35 (34.3)	1.63 (1.05–2.52)	0.027	
C	698 (75.7)	67 (65.7)			
Genotype					
TT	33 (7.2)	3 (5.9)	Reference		
TC	158 (34.3)	29 (56.9)	0.50 (0.14–1.72)	0.261	
CC	270 (58.6)	19 (37.3)	1.29 (0.36–4.60)	0.692	
Best model					
Recessive (CC vs. TC + TT)			2.38 (1.31–4.33)	0.004	
*KIF6* rs20455 (T > C)					0.401
T	630 (68.3)	65 (63.7)	0.81 (0.53–1.25)	0.345	
C	292 (31.7)	37 (36.3)			
Genotype					
TT	216 (46.9)	24 (47.1)	Reference		
TC	198 (43.0)	17 (33.3)	1.29 (0.68–2.48)	0.436	
CC	47 (10.2)	10 (19.6)	0.52 (0.23–1.17)	0.108	
Best model					
Recessive (CC vs. TC + TT)			0.47 (0.22–0.99)	0.043	

WHR—waist-to-hip ratio; CI—confidence interval; HW—Hardy–Weinberg; statistically significant at *p* < 0.05.

**Table 3:** Variables independently associated with elevated WHR.

Variables	B	S.E.	Wald	df	Odds Ratio (95% CI)	*p* Value
Diabetes	1.290	0.449	8.263	1	3.63 (1.51–8.75)	0.004
*SLC30A8* (CC)	0.917	0.308	8.876	1	2.50 (1.37–4.57)	0.003
Constant	1.499	0.204	53.926	1	4.475	<0.0001

WHR—waist-to-hip ratio; B—beta coefficient; S.E.—standard error; df—degrees of freedom; CI—confidence interval; statistically significant at *p* < 0.05. Variables excluded from the equation: age, smoking, hypertension, dyslipidaemia, BMI, physical inactivity, *PSRC1* (AA + GA), and *KIF6* (CC).

## Data Availability

All datasets generated during the current study are included in the main text or available in the online Supplementary Material.

## Supplementary Materials

The following supporting information can be downloaded at: https://www.mdpi.com/article/10.3390/genes16091019/s1, Table S1: The 15 genetic variants selected from the GENEMACOR Study, which are known to have a putative function in diabetes, obesity, or lipid metabolism [32,33,34,35,36,37,38,39,40,41,42,43,44,45,46].
