# Supplementary material for: Association of Obesity-Related Genetic Variants with Android Fat Patterning and Cardiometabolic Risk in Women
Source: Genes (Basel). 2025 Aug 28;16(9):1019. doi: 10.3390/genes16091019 (PMC12469512; doi:10.3390/genes16091019)
Supplement: Supplementary file 1 [file genes-16-01019-s001.zip › genes-3778277-supplementary.pdf]

Supplementary Material

Supplementary Table S1. The 15 genetic variants selected from the GENEMACOR Study, which are known to have a putative function in diabetes, obesity, or lipid metabolism

**SNP ID**	**Nearest Gene**	**Allele change**	**CHR**	**OR (Best model)**	**P value**	**MAF**	**Putative Function**	**References**
rs8050136	*FTO*	C>A	16	1.18$^{\#}$<br>1.02-1.37	0.026	39.7%	Insulin secretion<br>Obesity<br>Β cell function	(32)
rs2667293	*ADIPOQ*	C>G	3	1.14$^{\bullet}$<br>1.02-1.29	0.025	23.5%	Glucose& Lipid Metabolism<br>Obesity	(33)
rs1884613	*HNF4A*	C>G	20	1.13*<br>0.97-1.32	0.123	16.2%	Glucose & Lipid Metabolism<br>Obesity	(34)
rs1376251	*TAS2R50*	G>A	12	1.18*<br>0.62-2.25	0.606	11.9%	Glucose & Lipid Metabolism<br>Affect bitter taste	(35)
rs4402960	*IGF2BP2*	G>T	3	1.11*<br>0.87-1.41	0.385	30.5%	Glucose Tolerance<br>T2DM<br>Obesity	(36)
rs17782313	*MC4R*	T>C	18	1.37*<br>0.99-1.90	0.059	21.7%	Obesity	(37)
rs1326634	*SLC30A8*	T>C	8	1.06$^{\bullet}$<br>0.95-1.19	0.281	25.9%	Fasting Glucose<br>T2 DM	(38)
rs2114580	*PCSK9*	A>G	1	1.05*<br>0.91-1.21	0.473	26.4%	Lipid Metabolism	(39)
rs7412/rs429358[1]	*APOE*	Є4	19	1.27$^{\#}$<br>1.08-1.50	0.004	13.4%	Lipid Metabolism<br>Obesity	(40)
rs599839	*PSRC1*	G>A	1	1.15$^{\#}$<br>0.82-1.63	0.419	21.3%	Lipid Metabolism	(41)
rs20455	*KIF6*	T>C	6	1.17*<br>0.93-1.46	0.178	33.0%	Dyslipidemia<br>Obesity	(42)
rs3798220	*LPA*	T>C	6	1.45^{+}<br>1.19-1.76	<0.0001	2.0%	Lipid Metabolism<br>Obesity	(43)
rs964184	*ZNF259*	C>G	11	1.11^{+}<br>0.97-1.27	0.125	17.6%	Lipid and glucose Metabolism	(44)
rs1801282	*PPARG*	C>G	3	1.15$^{\bullet}$<br>0.97-1.38	0.110	8.7%	Obesity<br>Lipid & glucose Metabolism	(45)
rs7903146	*TCF7L2*	C>T	10	1.01$^{\#}$<br>0.82-1.25	0.924	35.2%	Obesity. T2 DM<br>Impairing β-cell function	(46)

*Chr – Chromosome; CI – Confidence Interval; $^{+}$Additive model;*Recessive model; $^{\#}$Dominant model; $^{\bullet}$Allelic model; [1]Resulting from a Haplotype. The table displays susceptibility loci for CAD, along with genotypic and allelic odds ratios (ORs) and p-values for the lead single nucleotide polymorphism (SNP) within each locus. Genotypic odds ratios (ORs) are provided for additive, recessive, allelic, and dominant models. The potential mechanism of action is based on what is already known about the function of the nearby genes.*
